# Subtype distribution of lymphomas in Southwest China: Analysis of 6,382 cases using WHO classification in a single institution

**DOI:** 10.1186/1746-1596-6-77

**Published:** 2011-08-22

**Authors:** Qun-Pei Yang, Wen-Yan Zhang, Jian-Bo Yu, Sha Zhao, Huan Xu, Wei-Ya Wang, Cheng-Feng Bi, Zhuo Zuo, Xiao-Qing Wang, Juan Huang, Lin Dai, Wei-Ping Liu

**Affiliations:** 1 Department of Pathology, West China Hospital of Sichuan University, Chengdu, China

**Keywords:** Distribution, Lymphoma, Subtype, WHO classification

## Abstract

**Background:**

The subtype distribution of lymphoid neoplasms in Southwest China was analyzed according to WHO classifications. This study aims to analyze subtype distribution of lymphomas in southwest China.

**Methods:**

Lymphoid neoplasms diagnosed within 9 years in a single institution in Southwest China were analyzed according to the WHO classification.

**Results:**

From January 2000 to December 2008, a total number of 6,382 patients with lymphoma were established, of which mature B-cell neoplasms accounted for 56%, mature T- and NK-cell neoplasms occupied 26%, and precursor lymphoid neoplasms and Hodgkin lymphomas were 5% and 13%, respectively. Mixed cellularity (76%) was the major subtype of classical Hodgkin lymphoma; and the bimodal age distribution was not observed. The top six subtypes of non-Hodgkin lymphoma were as follows: diffuse large B-cell lymphoma, extranodal NK/T-cell lymphoma, nasal type, extranodal marginal zone lymphoma of mucosa associated lymphoid tissue, follicular lymphoma, precursor lymphoid neoplasms, and chronic lymphocytic leukemia/small lymphocytic lymphoma. Extranodal lymphomas comprised about half of all cases, and most frequently involved Waldeyer's ring, gastrointestinal tract, sinonasal region and skin.

**Conclusions:**

The lymphoid neoplasms of Southwest China displayed some epidemiologic features similar to those reported in literature from western and Asian countries, as well as other regions of China, whereas some subtypes showed distinct features. The high frequency of mature T/NK cell neoplasms and extranodal lymphomas, especially for extranodal NK/T-cell lymphoma, nasal type, is the most outstanding characteristic of this series.

## Background

Lymphoid neoplasms are a diverse group of malignancy that originate from either B, T or NK cell, representing about 4% of the new cases of cancer diagnosed in China each year, making them the ninth most common cancer and the tenth leading cause of cancer death [1]. Previous epidemiologic studies have shown remarkable differences in the distribution of lymphoma subtypes between Asian and western populations [2-4]. However, the possible reasons of geographic differences in the spectrum of lymphoma remain unknown, mainly because the etiology of lymphoma is largely unknown, even though some risk factors have been documented recently, including genetic factors, abnormality of immunity, individual susceptibilities, lifestyles, environmental exposures, as well as various kinds of infections caused by bacteria, viruses, mycoplasma and chlamydia [5].

Major progression has been made in understanding the pathobiology of these diseases in the last two decades, leading to the development of the internationally adopted WHO classification system and its updated version [5,6]. From then on, many studies on lymphoma incidence patterns or distributions were reported all over the world. However, research on distribution of lymphoma in Mainland China was still limited, and the case number was also small. In the current study, 6,382 consecutive patients diagnosed with lymphoid neoplasms according to WHO classification in the last nine years were reviewed and analyzed. The purpose was to estimate the subtype distribution of lymphomas and major clinical features and to compare the data of ours with those reported in the literature.

## Patients and methods

The computerized database of all patients at the Department of Pathology, West China Hospital of Sichuan University was searched to identify patients with a diagnosis of lymphoma, excluding myeloid neoplasms and plasma cell myeloma/plasmacytoma. Cases in which the diagnoses of lymphoma were based on bone marrow biopsy alone and cases with inadequate materials were also excluded from this study. From January 2000 to December 2008, a total number of 6,382 cases diagnosed definitely with lymphoma were included. Clinical information including demographics (age, gender) and initial site involvement was collected from the submission of pathological samples and application tables for consultants.

Hematoxylin-eosin-stained sections were examined histopathologically. For immunohistochemical staining of paraffin-embedded sections, antibodies selected included CD3, CD3ε, CD4, CD5, CD8, CD10, CD15, CD20, CD21, CD23, CD30, CD43, CD45RO, CD56, CD79α, CD99, ALK-1 (anaplastic large cell lymphoma kinase-1), EMA (epithelial membrane antigen), cyclin D1, BCL-2, BCL-6, Mum1, κ and λ light chain, Ki-67, Granzyme B, TIA-1 (T-cell intracytoplasmic antigen-1), TdT, EBV. *In situ *hybridization for EBV-encoded small RNA (EBER) and IgH and/or TCR gene rearrangement detected by polymerase chain reaction were performed when the diagnosis was not clear from the histopathologic and immunophenotypic evaluation. All cases were diagnosed and classified in accordance with the criteria of WHO classification of Tumors of Haematopoietic and Lymphoid Tissue (2001). The protocol of this study was approved by the Institutional Review Board or ethical committee of West China Hospital of Sichuan University. Informed consent for the collection of medical information for in patients, out patients and consultants was obtained at their first visit.

## Results

A total number of 6,382 lymphomas were included in this study, in which 3,160 were consultant cases (49.5%) from all regions of southwestern China. All of the subtypes described in WHO classifications were presented but hairy cell leukemia, intravascular large B-cell lymphoma, primary effusion lymphoma, T-cell large granular lymphocytic leukemia, adult T-cell leukemia/lymphoma (ATLL) and both B- and T-cell prolymphocytic leukemia.

The major clinicopathologic characteristics of this series are shown in Table 1. Of the 6,382 lymphomas, 833 were Hodgkin lymphomas (HL) and 5,549 were non-Hodgkin lymphomas (NHL). Classical HL (CHL) accounted for 95.8% of HL. Three quarters were mixed cellularity (MC-CHL), and 18% were nodular sclerosis (NS-CHL). In all cases of 5,549 NHLs, 64.4% of them were mature B-cell neoplasms (3,571 cases). The most common subtype was diffuse large B-cell lymphoma (DLBCL), followed by extranodal marginal zone B-cell lymphoma of mucosa-associated lymphoid tissue (MALT lymphoma), follicular lymphoma (FL), chronic lymphocytic leukemia/small lymphocytic lymphoma (CLL/SLL), mantle cell lymphoma (MCL), and Burkitt lymphoma. In addition, 30.2% of NHLs were mature T- and NK-cell neoplasms (1,677 cases). The most common subtype was extranodal NK/T-cell lymphoma, nasal type (ENKTCL), followed by peripheral T-cell lymphoma, not otherwise specified (PTCL, NOS), anaplastic large cell lymphoma (ALCL), angioimmunoblastic T-cell lymphoma (AITL), subcutaneous panniculitis-like T-cell lymphoma (SPTCL) and primary cutaneous CD30 positive lymphoproliferative disorders (PCCD30LD). Furthermore, 5.4% of NHLs were lymphoblastic leukemia/lymphoma (LBL, 301 cases), most of which were T-lymphoblastic.

**Table 1:** Subtypes of lymphomas (n = 6,382)

		Age (year)		Sex			Site		
				
Subtype	**No**.	Median	Range	Male	Female	M:F	Nodal	Extranodal	N/E
**DLBCL**	2288	55	3-93	1359	929	1.5	1027	1261	0.81
**ENKTCL**	949	41	1-82	668	281	2.4	32	917	0.03
**MALT**	350	56	16-86	183	167	1.1	20	330	0.06
**FL**	327	54	23-83	213	114	1.9	292	35	8.34
**LBL**	301	19	1-71	211	90	2.3	253	48	5.27
**CLL/SLL**	256	60	22-88	180	76	2.4	228	28	8.14
**PTCL, NOS**	221	55	12-90	148	73	2	152	69	2.2
**ALCL**	196	27.5	4-71	118	78	1.5	151	45	3.36
**AITL**	185	61	20-86	124	61	2	185	0	-
**MCL**	175	61	20-79	132	43	3.1	126	49	2.57
**Burkitt**	106	15.5	2-69	78	28	2.8	49	57	0.86
**SPTCL**	54	33	4-68	25	29	0.9	0	54	0
**LPL**	44	61	30-82	25	19	1.3	34	10	3.4
**PCCD30LD**	37	37	2-78	18	19	0.9	0	37	-
**SMZBCL**	20	61.5	15-76	10	10	1	20	0	-
**HSTCL**	14	25	11-46	9	5	1.8	5	9	0.56
**MF/SS**	14	52.5	31-80	8	6	1.3	0	14	-
**ETCL**	7	29	17-42	5	2	2.5	0	7	-
**NMZBCL**	5	64	52-80	3	2	1.5	5	0	-
**MC-CHL**	606	31	3-89	433	173	2.5	597	9	66.3
**NS-CHL**	145	29	3-76	75	70	1.1	139	6	23.2
**LR-CHL**	35	30	4-64	29	6	4.8	35	0	-
**LD-CHL**	12	53	19-82	6	6	1	12	0	-
**NLPHL**	35	36	8-74	25	10	2.5	34	1	34

**Total**	**6382**	**50**	**1-93**	**4085**	**2297**	**1.8**	**3396**	**2986**	**1.1**

DLBCL, diffuse large B-cell lymphoma; ENKTCL, extranodal NK/T-cell lymphoma, nasal type; MALT, extranodal marginal zone lymphoma of mucosa associated lymphoid tissue; FL, follicular lymphoma; LBL, lymphoblastic leukemia/lymphoma; CLL/SLL, chronic lymphocytic leukemia/small lymphocytic lymphoma; PTCL, NOS, peripheral T-cell lymphoma, not otherwise specified; ALCL, anaplastic large cell lymphoma; AITL, angioimmunoblastic T-cell lymphoma; MCL, mantle cell lymphoma; Burkitt, Burkitt lymphoma; SPTCL, subcutaneous panniculitis-like T-cell lymphoma; LPL, lymphoplasmacytic lymphoma; PCCD30LD, primary cutaneous CD30 positive lymphoproliferative disorders; SMZBCL, splenic marginal zone B-cell lymphoma; HSTCL, hepatosplenic T-cell lymphoma; MF/SS, mycosis fungoides/Sezary syndrome; ETCL, enteropathy-associated T-cell lymphoma; NMZBCL, nodal marginal zone B-cell lymphoma; MC-CHL, mixed cellularity classical Hodgkin lymphoma; NS-CHL, nodular sclerosis classical Hodgkin lymphoma; LR-CHL, lymphocyte-rich classical Hodgkin lymphoma; LD-CHL, lymphocyte-depleted classical Hodgkin lymphoma; NLPHL, nodular lymphocyte predominant Hodgkin lymphoma.

Most subtypes of lymphomas were prone to involve males. Striking male predominance was observed for MCL and lymphocyte rich CHL (LR-CHL) with the M/F ratio of 3.1 and 4.8, respectively. For male patients, the top five common subtypes were as follows: DLBCL, ENKTCL, MC-CHL, FL and LBL; whereas for female patients, they were DLBCL, ENKTCL, MC-CHL, MALT and FL.

**Figure 1:**
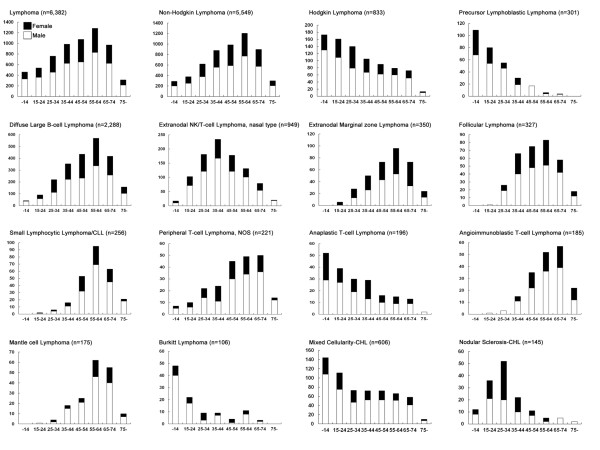
**Distribution of lymphoid neoplasms by subtype, age and sex**.

Age and sex distribution by subtypes of lymphoma is shown in Figure 1. The most common pediatric (age younger than 15 yr) lymphoma was MC-CHL, followed by LBL, ALCL, Burkitt, DLBCL. For adolescents and young adults (age 15 yr to 24 yr), MC-CHL was still the leading subtype, followed by ENKTCL, DLBCL, and LBL. For adults, DLBCL was the most common subtype, followed by ENKTCL, MC-CHL, FL, and MALT. For the elderly (aged older than 64 yr), DLBCL comprised approximately half of all lymphomas, followed by ENKTCL, MALT, CLL/SLL, and AITL.

**Figure 2:**
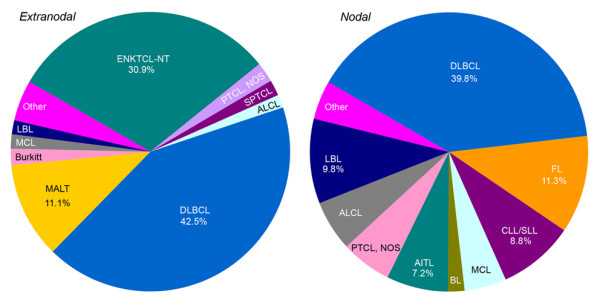
**Distribution of histological subtypes in extranodal and nodal lymphoma**.

**Figure 3:**
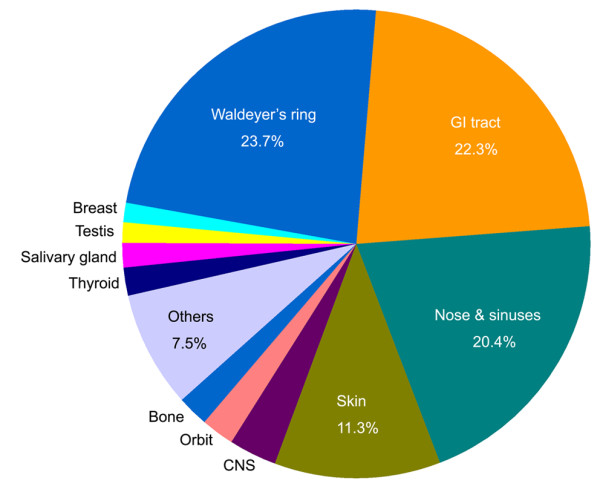
**Extranodal sites involved in non-Hodgkin lymphoma**. Waldeyer's ring includes tonsil, palate, nasopharynx, pharynx and base of tongue; GI tract (gastrointestinal tract) includes stomach, small intestine and colon; Nose & sinuses includes nasal cavity and paranasal sinuses; CNS, central nervous system.

In this study, extranodal lymphoma refers to lymphoma arising primarily from sites other than lymph nodes, spleen, bone marrow, or mediastinum (lymph nodes and thymus). Comparison of subtype distribution between extranodal and nodal lymphomas is shown in Figure 2. Approximately 53.5% of all NHL presented in extranodal sites at diagnosis. Figure 3 shows the anatomical sites most commonly involved by extranodal lymphomas. Lymphomas of Waldeyer's ring, gastrointestinal tract, sinonasal regions and skin represented most of the extranodal lymphomas. DLBCL was found to be the most common subtype of both extranodal and nodal lymphomas, followed by ENKTCL and FL, respectively. Concerning the distribution of histologic subtypes, those involving in particular extranodal sites, DLBCL and ENKTCL made up the majority of NHL in Waldeyer's ring; DLBCL and MALT composed over 80% of all lymphomas in the gastrointestinal tract; ENKTCL primarily represented the sinonasal lymphomas. For skin lymphomas, except for MF, PCCD30LPD, SPTCL and ENKTCL were relatively common. For nodal-based lymphomas, cervical, inguinal, axillary, and supraclavicular lymph nodes were frequently involved. About 76% of nodal-based lymphomas were NHLs, in which the top eight subtypes were as follows: DLBCL, FL, LBL, CLL/SLL, AITL, PTCL, NOS, ALCL, and MCL.

## Discussion

Epidemiologic studies suggest that distribution of lymphoma subtypes differs strikingly by geographic variations [2]. But there is limited information on this research in Mainland China. Up to now, this report demonstrates the largest comprehensive descriptive study of subtype distribution of lymphomas classified by the WHO criteria in a single institution from China.

According to the latest SEER data [7], HL made up 11% of all lymphomas. In our observation, this figure was 13%, similar to US and most of the reports from China but a little higher than Japan and South Korea [8-14]. As for subtypes, MC-CHL is the most common of HL, followed by NS-CHL in this series, whereas NS-CHL is much more frequent than MC-CHL (70% versus 20~25%) in Western countries [5,6,15]. MC-CHL has a strong association with EBV, and Armstrong *et al *[16] have proposed a three-disease model on the basis of age and EBV status. We have found that 73.3% of MC-CHL had EBV, whereas only one-fifth of NS-CHL cases had EBV and EBV was negative in the remaining subtypes. Therefore, EBV-associated disease in childhood may contribute to the high frequency of MC-CHL in the current study. However, more evidence will be needed. Another difference is that HL shows a gradual decline in cases with age in this series, whereas a bimodal age curve (a peak at 15~35 years of age and a second peak in late life) is apparent in Europe and North America. Furthermore, report from Japan shows that a single peak age of HL was in the elderly [10]. Nakatsuka and Aozasa [17] have pointed out that the bimodal age curve might be formed by the different peak ages of the two main subtypes, MC-CHL (later years) and NS-CHL (young adults).

Comparison on the incidence of NHL between our findings and others is shown in additional file 1, table 1. Similar to other reports, DLBCL was also the leading histological subtype, but the frequency of FL was lower than that of western countries, a phenomenon commonly seen in the Chinese population [18]. In China, DLBCL is still the most common subtype of B-cell lymphomas, but the second most common subtype varies a little from region to region [11-14,18-21]. Classification of DLBCL into prognostically distinct subtypes has progressed from gene expression profiles (GEP) in research to immunochemistry for a panel of markers which could be done routinely [22-26]. Based on Hans' algorithm [24], 79% of the 364 cases of DLBCL in which CD10, BCL-6 and Mum1 had been completely performed in this collection were categorized into non-GCB subtype, agreeing very well with the result of recent research for Chinese patients [27].

It is commonly thought that mature T/NK-cell neoplasms display higher rates on the Asian continent than others [28,29]. A recent large international retrospective study validated the geographic variations and showed the high frequency of ALCL, ALK-positive in North America, AITL and ETCL in Europe, ATLL in Japan and ENKTCL in Asian countries other than Japan [4]. In fact, the geographic variations also could be found across China; ENKTCL is the most common subtype of PTCL in Hong Kong and this group, whereas PTCL, NOS in all other parts [11-14,18-21,30]. Peripheral T/NK-cell lymphomas comprised 30.2% of non-Hodgkin lymphomas in the current study, higher than other Asian reports using WHO classification [8-14,18-21,30,31]. This is likely due to the strikingly high percentage of ENKTCL in this series. Research has suggested that environmental factors including EBV infection as well as exposure to pesticides and chemical solvents were strongly associated with this disease [32,33]. Considering that Sichuan province is a main agricultural area in China, this may be explained. However, further research on this aspect should be done. In addition, the high proportion of consultant cases (38.4%) may also contribute to the high percentage of ENKTCL.

As for age-specific incidence, the subtypes of pediatric (younger than 15-year old) lymphoma are limited, including CHL (mainly MC type), LBL, ALCL, Burkitt and DLBCL. This is in accordance with reports in the literature [34,35]. However, in any age group but pediatric, ENKTCL takes second place following DLBCL in this study. In addition, the median ages of most patients were about 10 years younger than that of Japanese and American patients [10,15]. The present findings may be due to an impressively increased and further increasing life expectancy and the high ratio of the aging population in developed countries [36].

The frequency of extranodal NHL varies in different parts of the world. Studies from Western countries have reported the occurrence of extranodal NHL as 24-48% of all NHL [37-40]. However, this figure is higher in Asia, for example, Pakistan (42%), Kuwait (45%), Japan (46.6%), Korea (55%), Thailand (58.7%), and China (44.9%-61.4%)[8-14,18-21,41-43]. The fluctuating frequency of extranodal lymphomas may be caused by genetic and ethnic factors, as well as the diverse definition criteria. Additionally, extranodal NHL in this series most commonly involved Waldeyer's ring, whereas the GI tract is reported to be the most common site in the literature [37-39]. The relatively high frequency of ENKTCL which mainly involves the sinonasal region and Waldeyer's ring may contribute to this difference.

Considering that the patients, including consultation cases, were referred from all regions of southwest China, the results may represent the distribution of lymphoma subtypes in southwest China. Diagnoses made on the bone marrow were excluded because this research was focused on lymphomas, not leukemia. This may result in under-representation of CLL and MALT lymphoma. However, since these diagnoses were not just based on bone marrow biopsy, the influence was not considerable.

In conclusion, subtype distribution of lymphomas in the current study is demonstrated and compared with reports all over the world and inside China. No bimodal age distribution was observed in CHL, and the major subtype of CHL is mixed cellularity, not nodular sclerosis. A high percentage of extranodal lymphomas are presented, including a relatively high frequency of ENKTCL.

## Competing interests

The authors declare that they have no competing interests.

## Authors' contributions

QPY participated in the design, analyses and data interpretation and drafted the manuscript. WYZ, JBY, SZ, HX, WYW, CFB, ZZ, XQW, JH, LD collected pathologic and clinical information and helped to draft the manuscript. WPL conceived of the study and participated in its design and analyses and helped to draft the manuscript. All authors read and approved the final manuscript.

## Supplementary Material

Additional file 1 **Subtype distribution of non-Hodgkin lymphoma across the world**. Subtype distribution of non-Hodgkin lymphoma across the world. Click here for file
