# Supplementary material for: Subtype distribution of lymphomas in Southwest China: Analysis of 6,382 cases using WHO classification in a single institution
Source: Diagn Pathol. 2011 Aug 22;6:77. doi: 10.1186/1746-1596-6-77 (PMC3179701; doi:10.1186/1746-1596-6-77)
Supplement: Additional file 1 — Subtype distribution of non-Hodgkin lymphoma across the world. Subtype distribution of non-Hodgkin lymphoma across the world. [file 1746-1596-6-77-S1.DOC]

**Additional file 1. Subtype distribution of non-Hodgkin lymphoma across the world**

| **Subtype** | **Present study** | **WHO**[28] | **USA**  [15] | **Korea**  [8] | **Japan**  [10] | **Thailand**  [9] | **China** | | | | | | |
| --- | --- | --- | --- | --- | --- | --- | --- | --- | --- | --- | --- | --- | --- |
|  |  |  |  |  |  |  | **HongKong**  [18] | **Taiwan**  [11] | **Shanghai**  [13] | **Shanxi**  [14] | **Beijing**  [20] | **Guangdong**  [19] | **Xinjiang**  [21] |
|  | **No.** | **No.** | **No.** | **No.** | **%^＊^** | **No.** | **No.** | **No.** | **No.** | **No.** | **No.** | **No.** | **No.** |
| **LBL-T** | 301 | 23 | 852 | 208 | 0.7 | 75 | - | 13 | 186 | 27 | 51 | 47 | 47 |
| **LBL-B** |  | - | 2907 | 602 | 0.2 | 16 | - | - | - | 4 | 7 | - | - |
| **DLBCL** | 2288 | 422 | 24246 | 1634 | 36.4 | 939 | 395 | 221 | 212 | 135 | 368 | 124 | 346 |
| **FL** | 327 | 304 | 10705 | 91 | 20.1 | 153 | 112 | 98 | 51 | 33 | 98 | 116 | 97 |
| **MALT** | 350 | 105 | 3247 | 661 | 4.6 | 76 | 82 | 34 | 18 | 45 | 69 | 31 | 94 |
| **CLL/SLL** | 256 | 93 | 16984 | 97 | 1.6 | 47 | 30 | 11 | 71 | 14 | 52 | 24 | - |
| **MCL** | 175 | 83 | 1691 | 98 | 3.0 | 19 | 30 | 24 | 14 | 10 | 6 | 2 | - |
| **Burkitt** | 106 | 39 | 1102 | 111 | 0.7 | 74 | - | 12 | 12 | 1 | 6 | 10 | - |
| **HCL** | - | - | 1116 | 5 | - | - | - | - | - | 1 | - | - | - |
| **LPL** | 44 | 16 | 2055 | 14 | 0.2 | 15 | - | 6 | 6 | 9 | 22 | 11 | - |
| **SMZBCL** | 20 | 11 | - | 5 | 0.4 | - | - | - | 5 | 1 | 3 | 2 | - |
| **DLBCL-m** | - | 33 | - | 16 | 0.4 | 16 | 30 | 12 | - | 1 | 59 | - | - |
| **NMZBCL** | 5 | 25 | - | 54 | 1.5 | - | 30 | 6 | 8 | 2 | 3 | 11 | - |
| **ENKTCL** | 949 | 19 | 0 | 235 | 1.7 | - | 58 | 17 | 13 | 5 | 110 | 16 | 62 |
| **PTCL, NOS** | 221 | 53 | 2532**^＊＊^** | 211 | 5.0 | 240 | 24 | 23 | 40 | 46 | 137 | 59 | 185 |
| **ALCL** | 196 | 33 | 864 | 104 | 2.2 | 67 | 25 | 8 | 15 | 16 | 32 | 11 | - |
| **AITL** | 185 | 17 | 176 | 43 | 5.7 | 33 | 19 | 3 | 26 | 9 | 24 | 4 | - |
| **ATLL** | - | 1 | - | 1 | 11.0 | - | - | - | - | 3 | - | - | - |
| **SPTCL** | 54 | - | - | 17 | - | 9 | 1 | 3 | - | 1 | 5 | 4 | - |
| **PCCD30LD** | 37 | - | - | 8 | - | 5 | 12 | 2 | - | 3 | - | - | - |
| **MF/SS** | 14 | 11 | 1773 | 21 | 0.5 | 23 | 4 | 2 | - | 2 | - | 3 | - |
| **HSTCL** | 14 | 1 | - | 3 | - | - | 1 | 1 | - | - | - | - | - |
| **ETCL** | 7 | 5 | - | 17 | - | - | 3 | 6 | - | 5 | - | 3 | - |
| **Others** | - | 84 | 7240 | 81 | 3.0 | 19 | 37 | 8 | 30 | 4 | 74 | 11 | 181 |
| **Total** | **5549** | **1378** | **77490** | **4337** | **2260** | **1826** | **893** | **510** | **707** | **377** | **1125** | **489** | **1012** |

**^＊^**The percentage is used in order to be accordant with the primary data.

**^＊＊^**In the primary report, this figure includes Peripheral T-cell lymphoma, NOS and T/NK-cell lymphoid neoplasms, NOS.

LBL-T, T lymphoblastic leukemia/lymphoma; LBL-B, B lymphoblastic leukemia/lymphoma; DLBCL, diffuse large B-cell lymphoma; FL, follicular lymphoma; MALT, extranodal marginal zone lymphoma of mucosa associated lymphoid tissue; CLL/SLL, chronic lymphocytic leukemia/small lymphocytic lymphoma; MCL, mantle cell lymphoma; Burkitt, Burkitt lymphoma; HCL, hairy cell leukemia; LPL, lymphoplasmacytic lymphoma; SMZBCL, splenic marginal zone B-cell lymphoma; DLBCL-m, primary mediastinal/thymic large B-cell lymphoma; NMZBCL, nodal marginal zone B-cell lymphoma; ENKTCL, extranodal NK/T-cell lymphoma, nasal type; PTCL, NOS, peripheral T-cell lymphoma, not otherwise specified; ALCL, anaplastic large cell lymphoma; AITL, angioimmunoblastic T-cell lymphoma; ATLL, adult T-cell leukemia/lymphoma; SPTCL, subcutaneous panniculitis-like T-cell lymphoma; PCCD30LD, primary cutaneous CD30 positive lymphoproliferative disorders; MF/SS, mycosis fungoides/Sezary syndrome; HSTCL, hepatosplenic T-cell lymphoma; ETCL, enteropathy-associated T-cell lymphoma.
